# Lung injury induced by different negative suction pressure in patients with pneumoconiosis undergoing whole lung lavage

**DOI:** 10.1186/s12890-022-01952-w

**Published:** 2022-04-22

**Authors:** Mingyuan Yang, Baoping Li, Bin Wang, Lei Li, Yurong Ji, Yunzhi Zhou, Rui Huang, Qinghao Cheng

**Affiliations:** 1 Center of Anesthesiology and Pain, Emergency General Hospital, Beijing, 100028 China; 2 Occupation Medicine Department, Emergency General Hospital, Beijing, 100028 China; 3 Department of Obstetrics and Gynecology, Emergency General Hospital, Beijing, 100028 China

**Keywords:** Pneumoconiosis, Whole lung lavage, Negative suction pressure, Lung injury

## Abstract

**Background:**

Pneumoconiosis is a diffuse interstitial fibronodular lung disease, which is caused by the inhalation of crystalline silica. Whole lung lavage (WLL) is a therapeutic procedure used to treat pneumoconiosis. This study is to compare the effects of different negative pressure suction on lung injury in patients with pneumoconiosis undergoing WLL.

**Materials and methods:**

A prospective study was conducted with 24 consecutively pneumoconiosis patients who underwent WLL from March 2020 to July 2020 at Emergency General Hospital, China. The patients were divided into two groups: high negative suction pressure group (group H, n = 13, negative suction pressure of 300–400 mmHg) and low negative suction pressure group (group L, n = 11, negative suction pressure of 40–50 mmHg). The arterial blood gas, lung function, lavage data, oxidative stress, and inflammatory responses to access lung injury were monitored.

**Results:**

Compared with those of group H, the right and left lung residual were significantly increased in the group L (*P* = 0.04, *P* = 0.01). Potential of hydrogen (pH), arterial partial pressure of oxygen (PaO_2_), arterial partial pressure of carbon dioxide (PaCO_2_), lactic acid (LAC) and glucose (GLU) varied from point to point in time (*P* < 0.01, *respectively*). There was statistical difference in the trend of superoxide dismutase (SOD) and interleukin-10 (IL-10) over time between the two groups (*P* < 0.01, *P* = 0.02). In comparison with the group H, the levels of IL-10 (*P* = 0.01) and SOD (*P* < 0.01) in WLL fluid were significantly increased in the group L. There was no statistical difference in the trend of maximal volumtary ventilation (MVV), forced vital capacity (FVC), forced expiratory volume in one second (FEV1%), residual volume (RV), residual volume/total lung capacity (RV/TLC), carbon monoxide dispersion factor (DLCO%), forced expiratory volume in one second/ forced vital capacity (FEV1/FVC%) over time between the two groups (*P* > 0.05, *respectively*).

**Conclusion:**

Low negative suction pressure has the potential benefit to reduce lung injury in patients with pneumoconiosis undergoing WLL, although it can lead to increased residual lavage fluid. Despite differing suction strategies, pulmonary function parameters including FEV1%, RV and DLCO% became worse than before WLL.

*Trial Registration* Chinese Clinical Trial registration number ChiCTR2000031024, 21/03/2020.

## Introduction

Whole lung lavage (WLL) is a therapeutic procedure used to treat pneumoconiosis and silicosis [1]. Pneumoconiosis is a systemic disease with main manifestation of pulmonary diffuse fibrosis caused by long-term inhalation and deposition of occupational dust containing silicon dioxide (SiO_2_)[2]. WLL can relieve respiratory symptoms, prolong life and improve quality of life by removing dust, fibrosis factors, lipid protein, inflammatory cells in alveolar cavity and bronchial tree [3]. For early pneumoconiosis patients, WLL can prevent further development of pneumoconiosis and significantly improve the long-term prognosis of the patients [4]. WLL had been regarded as the most effective treatment for early pneumoconiosis.

WLL was performed under general anesthesia with lung separation obtained by a double-lumen endobronchial tube (DLT). While mechanical ventilation was maintained in one lung, the contralateral lung were repeatedly filled with saline and then drained by gravity [5, 6]. The complications of WLL included hypoxemia, loss of lung isolation, hydrothorax and pneumothorax [1]. If complications were not properly treated in time, WLL effect would be affected and even life safety of patients would be endangered.

The key technique of bilateral massive WLL was how to reduce residual pulmonary fluid and restore diffuse pulmonary function after each side lung lavage. Previous reviews reported the removal of lavage fluid from the lungs by gravity, may result in large amounts of residual fluid, longer drainage time, and even inability to lavage bilateral lungs simultaneously [7]. The deficit between the volume of lavage infusion and drain under gravity is larger [8], with more than 10% [9]. In this study, negative suction was used to remove the lavage fluid of lungs, which was divided into two different degrees. However, the effect of two different negative suction pressures on lung injury, pulmonary lavage characteristics, lung recovery time and pulmonary function during WLL was not clear. Specially, we evaluated the hypothesis that if low pressure negative suction may have the potential benefits to decrease lung injury and improve pulmonary function in patients with pneumoconiosis undergoing WLL.

## Materials and methods

### Study design and patient objectives

This was a single center, randomized controlled trial to assess the effect of two different negative suction pressures on lung injury, pulmonary lavage status and pulmonary function in patients with pneumoconiosis undergoing WLL.

The protocol was approved by the ethics committee of Emergency General Hospital, China. Written consent was obtained from each participant. This study was registered in Chinese Clinical Trial Registry on March 21, 2020 (Registration number: ChiCTR2000031024). It also followed the Consolidated Standards of Reporting Trials (CONSORT) guidelines.

A total of 24 pneumoconiosis patients who underwent WLL from May 2020 to July 2020 were enrolled in this study. The inclusion criteria were (i) 18 years < age < 80 years; (ii) Sex unlimited; (iii) Pre-operative forced expiratory volume in one second (FEV1%) ≥ 65%; (iv) Pre-operative arterial partial pressure of oxygen (PaO_2_) > 70 mmHg; (v) According to epidemiological and occupational history, the patients were diagnosed as pneumoconiosis by X-ray, and needed bilateral lung lavage at the same time. The exclusion criteria were (i) Patients who underwent WLL within one year; (ii) Patients with severe tracheobronchial malformations that prevented the double lumen trachea catheter from being in place; (iii) Patients with acute respiratory tract infection and had not been cured; (iv) Patients with bullae whose diameter of subpleural were more than 2 cm; (v) Patients with severe emphysema; (vi) Patients with coagulation dysfunction; (vii) Patients with uncontrolled hypertension; (viii) Patients with leukopenia.

**Fig. 1:**
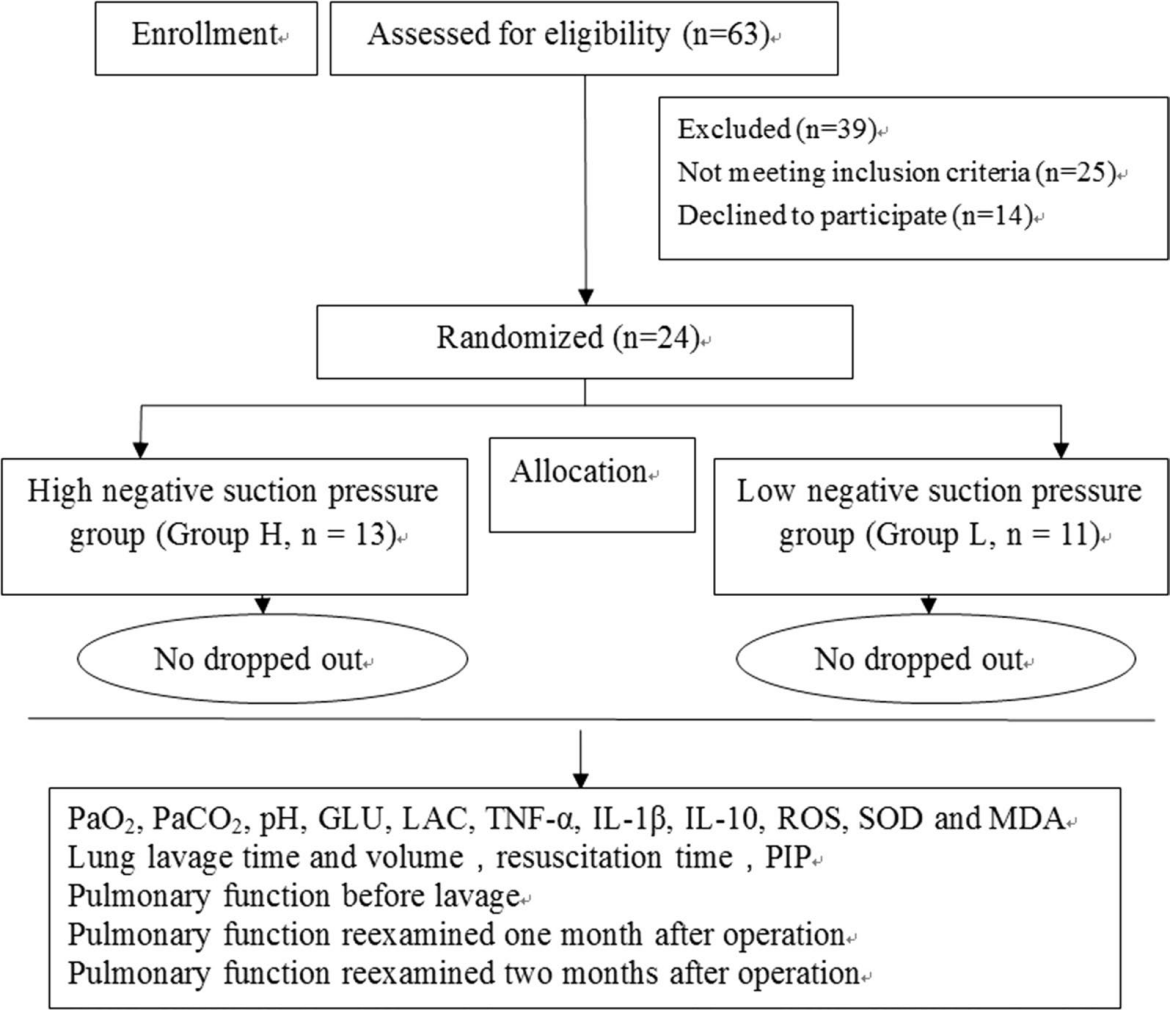
Trial flow diagram

The patients were randomly divided into two groups: high negative suction pressure group (group H, n = 13, negative suction pressure of 300-400 mmHg) and low negative suction pressure group (group L, n = 11, negative suction pressure of 40-50 mmHg). Group allocation number was placed in an envelope. The negative suction pressure regulation, sample collection, clinical follow-ups and laboratory research were carried out by different researchers who were all blind to the grouping situation. The patients and surgeons were all blind to the grouping situation. The details were as shown Fig. 1.

### Anesthetic settings and maintenance

Standardized approach of anesthesia induction was performed and left DLT was inserted in place. Intraoperative maintenance: propofol 4 ~ 8 mg kg^−1^·h^−1^, remifentanil 0.15 ~ 0.3 μg·kg^−1^·min^−1^, intermittent administration of rocuronium 0.2 ~ 0.4 mg·kg^−1^ to maintain anesthesia depth and muscle relaxation.

After the DLT was placed, bilateral lung isolation was judged by clinical signs, and then determined by auscultation and fiberoptic bronchoscopy [10]. The parameters of the ventilator were set at 12 ~ 14 breaths/min, with volume of 8 ~ 10 ml/kg, I:E = 1:2, and oxygen concentration of 100%. The parameters of one-lung ventilation were set at 14 ~ 18 breaths/min, with a volume of 6 ~ 8 ml·kg^−1^ at inspired oxygen concentration of 100%. Airway pressure was maintained < 50 cm H_2_O.

### The procedures of whole lung lavage

The infusion tube and the drainage tube were connected to one side of the DLT through a Y-tube. The lavage fluid was infused by gravity, and suspended at the level of 40 cm above the axillary midline. The lavage fluid was drained out by suction apparatus. Negative suction pressure is set to 300-400 mmHg or 40-50 mmHg. The lavage fluid was saline at 37℃. The order of lavage was right lung followed by left lung during the procedure. At the end of lavage, the negative pressure attraction should be thorough to reduce the residual liquid in the lavage side lung as much as possible. When the patient woke up and recovered spontaneous breathing, the DLT tube was removed, and then the patient was sent back to ward.

### Observe indicators and assessment

Demographic and clinical characteristics of study population in both groups were recorded. Arterial blood gas (ABG) analysis at T1-before anesthesia induction, T2-before left lung lavage, T3-at the end of operation when bilateral lung ventilation recovery and T4-six hours after operation were recorded. Lung lavage time and volume of lavage fluid in both groups were recorded during operation.

Serum was collected at T1, T3 and T5- the third day after operation, and lavage fluid was collected at T3. The concentration of inflammation and the oxidation factors in them were measured by enzyme-linked immunosorbent assay (ELISA). Inflammation and the oxidation factors include tumor necrosis factor-α (TNF-α), interleukin-1β (IL-1β), interleukin-10 (IL-10), reactive oxygen species (ROS), malondialdehyde (MDA) and superoxide dismutase (SOD).

The pulmonary function parameters in both groups before operation (T1), one month after operation (T1-mon) and two months after operation (T2-mon) were also recorded, including maximal volumtary ventilation (MVV); forced vital capacity (FVC); FEV1%; residual volume (RV); total lung capacity (TLC); carbon monoxide dispersion factor (DLCO%); forced expiratory volume in one second/ forced vital capacity (FEV1/FVC%).

### Statistical analysis

The sample size was estimated by the formula of n = (μ_α_ + μ_β_)^2^σ^2^/δ^2^ with a standard deviation of 0.8, and bilaterally equal to 0.05, or even 0.2 (power = 0.8). The concentration of inflammation and oxidation factors in serum and WLL fluid were the main primary and secondary outcome. The results were expressed as mean ± S.D, or the number and percentage. SPSS 20.0 software was used for data collation and statistical analysis. Parameters changes over time from baseline within each group were determined by repeated measures univariate analysis of variance (ANOVA). Differences between the groups at each time point were evaluated by 1-way ANOVA. Enumeration data was tested by χ^2^ or Fisher precision test. A value of *P* < 0.05 was considered statistically significant.

## Results

The characteristics of enrolled subjects were summarized in Table 1. Significant differences in sex ratio, age, body massive index (BMI), stage of pneumoconiosis, smoke history, or dust exposure years between both groups were not observed.

**Table 1:** Demographic and clinical characteristics of study population in both groups

Variables	Group H (n = 13)	Group L (n = 11)	*P*
Male (%)	13(100.0)	11(100.0)	1
Age (years)	48.5 ± 5.9	45.2 ± 6.7	0.21
BMI	25.4 ± 2.1	23.6 ± 4.0	0.18
Stage of pneumoconiosis I and II (%)	8(61.5)	9(81.8)	0.39
Stage of pneumoconiosis III (%)	5(38.5)	2(18.2)	0.39
Smoke history (%)	9(69.0)	6(54.5)	0.68
Dust exposure time(year)	17.8 ± 9.8	16.0 ± 11.0	0.72

Measurement data are expressed as means ± SD. Counting data were expressed as numbers and percentages. *was statistically significant compared with Group H, *P* < 0.05

Group H, high negative suction pressure group; Group L, low negative suction pressure group

*BMI* body mass index

Lung lavage status of enrolled subjects was summarized in Table 2. There were no significant differences in right lung lavage time, right lung resuscitation time, left lung lavage time, left lung resuscitation time, right and left lung lavage volume, peak inspiratory pressure (PIP) at right lung lavage time, left lung lavage time and bilateral lung ventilation between both groups. Compared with group H, right and left lung residual volume were significantly increased in the group L (*P* = 0.04, *P* = 0.01).

**Table 2:** Lung lavage time and volume of lavage fluid in both groups

Variables	Group H (n = 13)	Group L (n = 11)	*P*
Right lung lavage time (min)	56.2 ± 15.3	65.0 ± 14.7	0.16
Right lung resuscitation time (min)	18.5 ± 5.2	17.7 ± 3.4	0.69
Left lung lavage time (min)	57.3 ± 15.4	62.7 ± 11.9	0.35
Left lung resuscitation time (min)	21.5 ± 4.7	20.0 ± 5.5	0.47
PIP (right lung lavage time)	26.0 ± 7.5	29.3 ± 8.3	0.40
PIP (left lung lavage time)	36.9 ± 5.7	35.4 ± 6.5	0.62
PIP (bilateral lung ventilation)	28.8 ± 7.4	27.5 ± 6.6	0.71
Right lung lavage volume (ml)	9625.0 ± 1060.7	8700.0 ± 1059.4	0.08
Right lung residual volume (ml)	550.0 ± 207.0	785.0 ± 221.2*	**0.04**
Left lung lavage volume (ml)	9500.0 ± 1069.0	8400.0 ± 1173.8	0.05
Left lung residual volume (ml)	425.0 ± 225.2	780.0 ± 278.1*	**0.01**

Measurement data are expressed as means ± SD. Counting data were expressed as numbers and percentages. *was statistically significant compared with Group H, *P* < 0.05. Compared with group H, right and left lung residual volume were significantly (bold) increased in the group L (*P* = 0.04, *P* = 0.01)

Group H, high negative suction pressure group; Group L, low negative suction pressure group

*PIP* peak inspiratory pressure

**Fig. 2:**
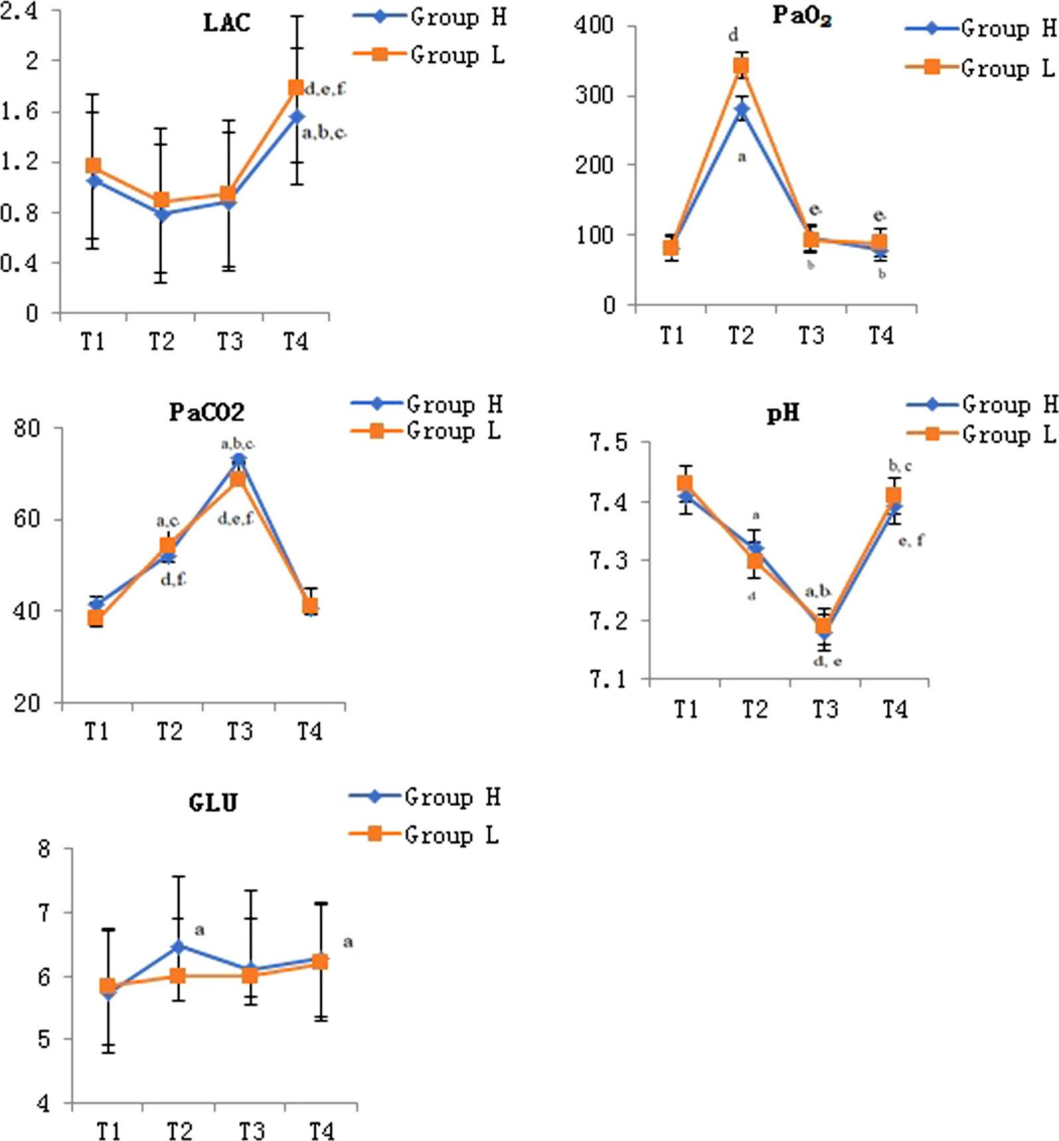
Arterial blood gas analysis of both groups at four time points. *Was statistically significant compared with Group H, *P* < 0.05; ^a^Was statistically significant compared with T1 in the group H, *P* < 0.05; ^b^Was statistically significant compared with T2 in the group H, *P* < 0.05; ^c^Was statistically significant compared with T3 in the group H, *P* < 0.05; ^d^Was statistically significant compared with T1 in the group L, *P* < 0.05; ^e^Was statistically significant compared with T2 in the group L, *P* < 0.05; ^f^Was statistically significant compared with T3 in the group L, *P* < 0.05; T1, before anesthesia induction; T2, before left lung lavage; T3, at the end of operation when bilateral lung ventilation recovery; T4, six hours after operation

ABG analysis results were shown in Fig. 2. There was no statistical difference in the trend of potential of hydrogen (pH), PaO_2_, arterial partial pressure of carbon dioxide (PaCO_2_), lactic acid (LAC) and glucose (GLU) over time between the two groups (*P* = 0.57, *P* = 0.51, *P* = 0.38, *P* = 0.62, *P* = 0.27), while which would vary from point to point in time (*P* < 0.01, *respectively*). The pH of group H and group L at T2 and T3 was significantly lower than those at T1 (*P* < 0.01, *respectively*). The pH of group H and group L at T3 was significantly lower than those at T2 (*P* < 0.01, *P* < 0.01). The pH of group H and group L at T4 was significantly higher than those at T2 and T3 (*P* < 0.01, *P* < 0.01, *respectively*). The PaO_2_ of group H and group L at T2 was significantly higher than those at T1 (*P* < 0.01, *P* < 0.01). The PaO_2_ of group H and group L at T3 and T4 was significantly lower than those at T2 (*P* < 0.01, *respectively*). The PaCO_2_ of group H and group L at T2 and T3 was significantly higher than those at T1 and T4 (*P* < 0.01, *respectively*). The PaCO_2_ of group H and group L at T3 was significantly higher than those at T2 (*P* < 0.01, *P* = 0.03). The LAC of group H and group L at T4 was significantly higher than those at T1, T2 and T3 (*P* < 0.01, *respectively*). The GLU of group H at T2 and T4 was significantly higher than that at T1 (*P* = 0.03, *P* < 0.01).

**Fig. 3:**
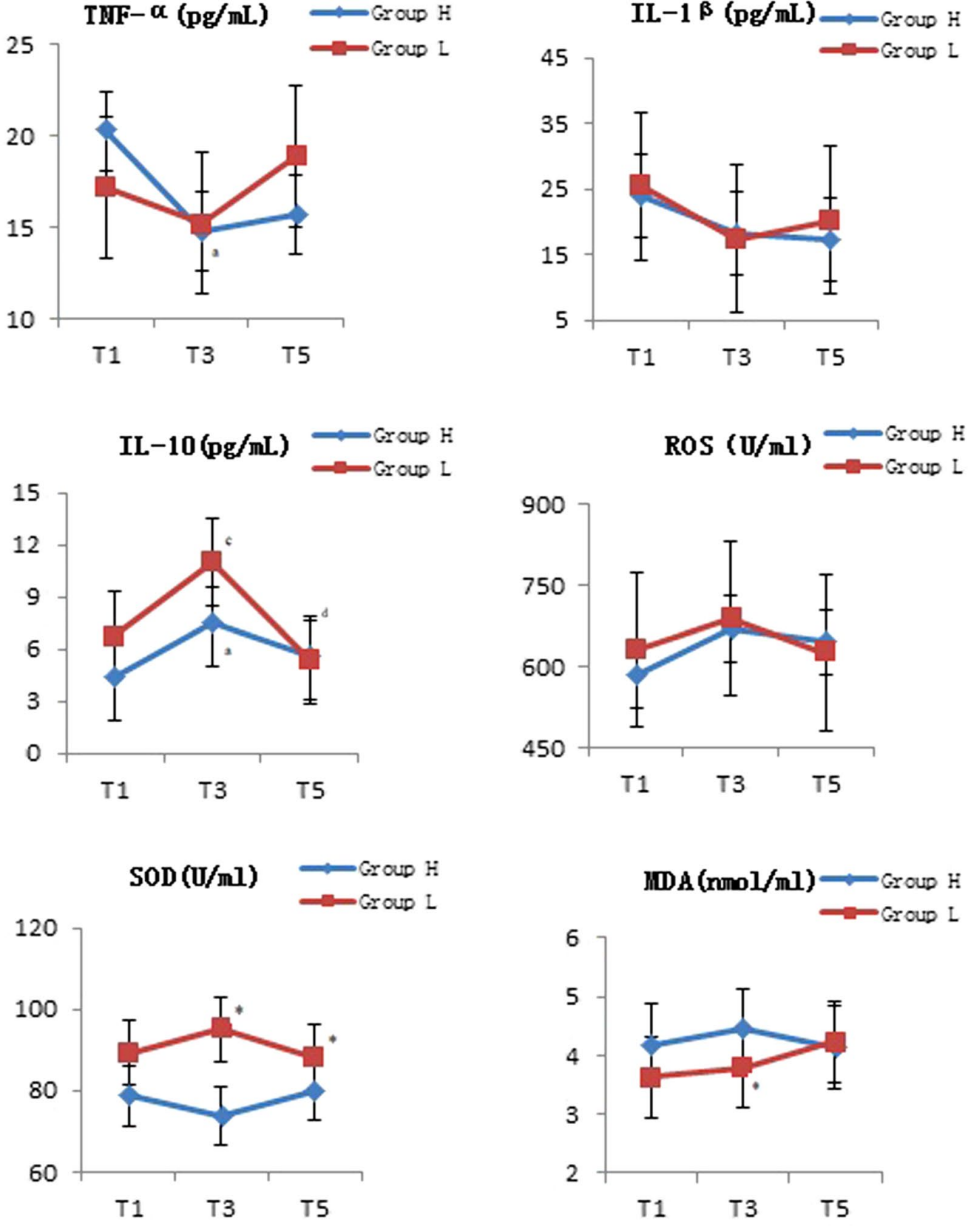
Effect of serum index on inflammation and oxidation system of both groups. *Was statistically significant compared with Group H, *P* < 0.05; ^a^was statistically significant compared with T1 in the group H, *P* < 0.05; ^b^was statistically significant compared with T3 in the group H, *P* < 0.05; ^c^was statistically significant compared with T1 in the group L, *P* < 0.05; ^d^was statistically significant compared with T3 in the group L, *P* < 0.05; T1, before anesthesia induction; T3, at the end of operation when bilateral lung ventilation recovery; T5, the third day after operation

There was no statistical difference in the trend of IL-1β, ROS and MDA over time between the two groups (*P* = 0.66, *P* = 0.64, *P* = 0.07), while which would not vary from point to point in time either (*P* = 0.40, *P* = 0.12, *P* = 0.45). However, the level of MDA in group H was significantly higher than that in group L (*P* < 0.01). There was statistical difference in the trend of SOD over time between the two groups (*P* < 0.01), which would not vary from point to point in time (*P* = 0.97). The serum levels of SOD in group L were significantly higher than that in group H at T3 and T5 (*P* < 0.01, *P* = 0.01). There was no statistical difference in the trend of TNF-α over time between the two groups (*P* = 0.05), while which would vary from point to point in time (*P* = 0.01). The serum levels of TNF-α in group H at T3 was significantly lower than that at T1 (*P* < 0.01). There was statistical difference in the trend of IL-10 over time between the two groups (*P* = 0.02), which would also vary from point to point in time (*P* < 0.01). The serum levels of IL-10 in group H and group L at T3 were significantly higher than those at T1 (*P* = 0.04, *P* < 0.01). The serum levels of IL-10 in group L at T5 was significantly lower than that at T3 (*P* < 0.01). In comparison with the group H, the levels of IL-1β, TNF-α, ROS and MDA in WLL fluid were not significantly changed, while IL-10 (*P* = 0.01) and SOD (*P* < 0.01) were significantly increased in the group L. The details were show in Fig. 3 and Table 3.

**Table 3:** Variables in WLL fluid on inflammation and the oxidation system

Variables in WLL fluid	Group H (n = 13)	Group L (n = 11)	*P*
TNF-α (pg·ml^−1^)	10.96 ± 3.04	10.76 ± 2.44	0.36
IL-1β (pg·ml^−1^)	14.09 ± 9.16	11.08 ± 5.91	0.87
IL-10 (pg·ml^−1^)	0.72 ± 0.42	1.58 ± 1.03*	**0.01**
ROS (U·ml^−1^)	561.16 ± 76.33	517.92 ± 122.40	0.30
MDA (nmol·ml^−1^)	3.64 ± 0.97	3.40 ± 0.33	0.50
SOD (U·ml^−1^)	56.64 ± 23.65	82.04 ± 7.14*	** < 0.01**

Measurement data are expressed as means ± SD. *Was statistically significant compared with Group H, *P* < 0.05

Group H, high negative suction pressure group; Group L, low negative suction pressure group

The levels of TNF-α, IL-1β and IL-10 in WLL fluid were detected by using rat-specific enzyme-linked immunosorbent assay (ELISA) kits. Levels of ROS, MDA, and SOD in WLL fluid were measured by using commercial kits

*WLL* whole lung lavage; *TNF-α* tumor necrosis factor-α; *IL-1β* interleukin-1β; *IL-10* interleukin-10; *ROS* reactive oxygen species; *MDA* malondialdehyde; *SOD* superoxide dismutase

**Fig. 4:**
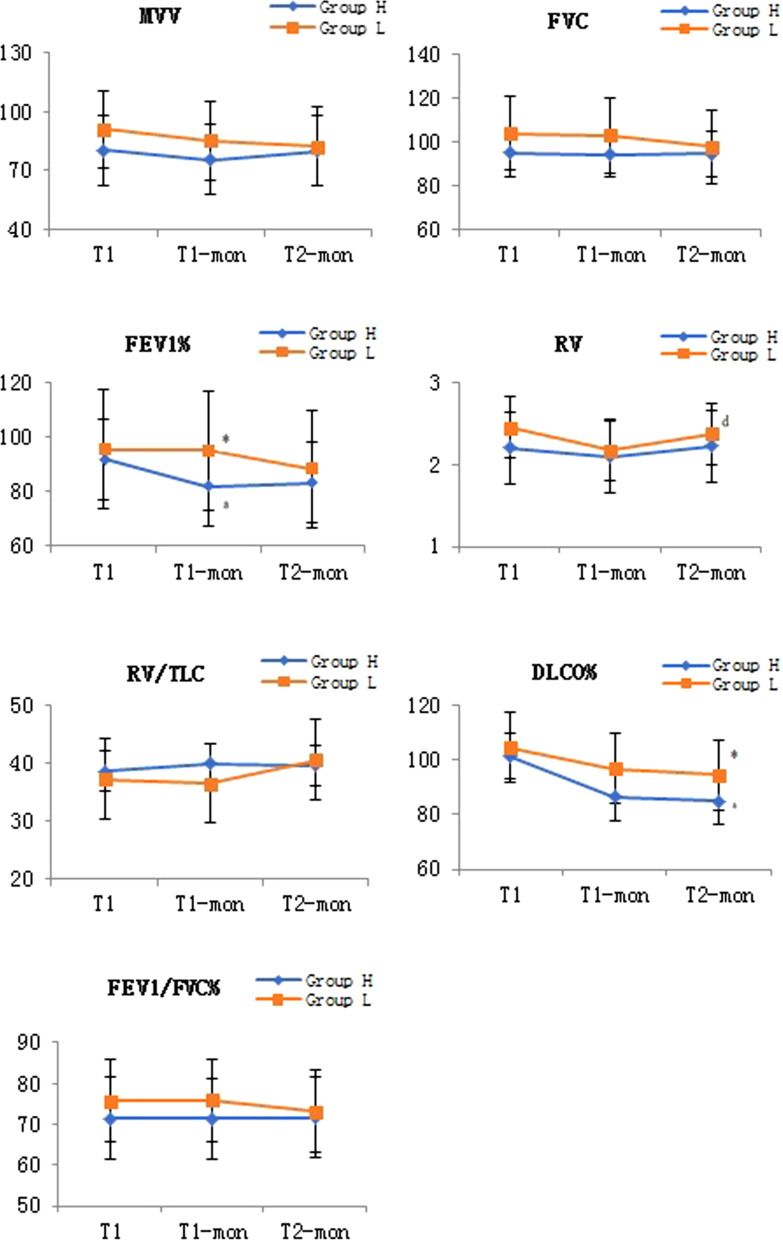
Effect of pulmonary function parameters  of both groups. *Was statistically significant compared with Group H, *P* < 0.05; ^a^was statistically significant compared with T1 in the group H, *P* < 0.05; ^b^was statistically significant compared with T1-mon in the group H, *P* < 0.05; ^c^was statistically significant compared with T1 in the group L, *P* < 0.05; ^d^was statistically significant compared with T1-mon in the group L, *P* < 0.05. T1, before operation; T1-mon, one month after operation; T2-mon, two months after operation

There was no statistical difference in the trend of MVV%, FEV1%, RV and DLCO% over time between the two groups (*P* = 0.48, *P* = 0.25, *P* = 0.71, *P* = 0.62, *P* = 0.71), while which would vary from point to point in time (*P* = 0.02, *P* = 0.04, *P* < 0.01, *P* = 0.02). There was no statistical difference in the trend of FVC, RV/TLC and FEV1/FVC% over time between the two groups (*P* = 0.52, *P* = 0.18, *P* = 0.34), while which would not vary from point to point in time either (*P* = 0.10, *P* = 0.31, *P* = 0.47). FEV1% in group L was significantly better than group H at T1-mon (*P* = 0.04). FEV1% in group H at T1-mon was significantly worse than that at T1 (*P* < 0.01). RV in group L at T2-mon was significantly higher than that at T1-mon (*P* < 0.01). DLCO% in group L was significantly higher than group H at T2-mon (*P* = 0.03). While DLCO% in group H T2-mon was significantly lower than at T1 (*P* = 0.02). The details were shown in Fig. 4.

## Discussion

Pneumoconiosis is the leading cause of invalidity among occupational respiratory diseases and associated to serious complications which could lead to death. There is no specific treatment for pneumoconiosis, and current therapy mainly centers on preventing complications, eliminating continued exposure to silica dust. The process of pneumoconiosis and chronic respiratory failure cannot be prevented even in isolation from the working environment. Several drugs and procedures, such as WLL, have been suggested. WLL is the effective treatment modality of choice for severe pneumoconiosis. Since its first description in 1967 by Ramirej, WLL is the gold standard treatment modality for silicosis [7, 11]. The aim of WLL is to remove excessive occupational dust material from the alveoli. Lung transplant is a potential option for the final stage of this pathology and should seriously be considered in the appropriate clinical context [12]. Davis summarized the research on the pathogenesis of silicosis in recent 30 years, and proposed that free SiO_2_ and polyacrylamide alveolar macrophage (PAM) were the main pathogenic factors of silicosis [13]. The interaction between them was the key to the pathogenesis of silicosis. Studies had confirmed that WLL can not only clear SiO_2_ and PAM in alveoli but also in pulmonary interstitial. Therefore, the theoretical basis of WLL in treating silicosis is consistent with the pathogenesis of modern silicosis.

WLL was performed using DLT and one lung ventilation (OLV) anesthesia. WLL in severely ill and young children may be performed with the assistance of cardiopulmonary bypass (CPB) [14]. OLV during WLL induces severe desaturation with fatal consequences. The risk of hypoxemia is even greatest during the drainage of lavage fluid due to shunting of blood through the non-ventilated lung [11]. OLV also has adverse effects on the respiratory system, may cause or aggravate lung injury and affect patient prognosis after surgery [15]. Patients with pneumoconiosis are more likely to suffer lung injury due to decreased lung compliance and pulmonary function.

Traditional WLL was to extract lavage fluid by gravity [7]. In this study negative suction was used to remove the lavage fluid of lungs. Different levels of negative pressure suction had different effects against acute lung injury caused by WLL. Lung injury can lead to refractory hypoxemia and respiratory failure which may result in an unplanned intensive care unit admission [16]. Lavage fluid absorption during procedures can lead to pulmonary edema and exacerbate respiratory insufficiency. The variation trend of intraoperative blood gas in two groups was consistent. Due to lung lavage, PaCO_2_ increased, PaO_2_ and the pH decreased significantly, which were recovered after the WLL. The intraoperative lavage time, resuscitation time and PIP of the two groups were not affected by the change of negative pressure suction pressure, and the oxygenation function of the patients during and after the operation were not affected either. The results showed that right and left lung residual fluid in group L were significantly increased compared with group H. While increased residual lung volume in group L may aggravate postoperative pulmonary edema. Poor lung compliance, poor pulmonary function, and a large amount of lavage fluid residual in the lungs made the intraoperative airway pressure increasing sharply, thus aggravating lung injury.

The robust oxidative stress is a major initiating factor of acute lung injury caused by WLL [17, 18]. Reactive oxygen species and inflammation factors play key role and mediates inflammation in acute respiratory distress syndrome patients [19]. MDA is a marker of the damage caused by oxidative stress, while SOD levels are usually used to evaluate primary defenses against cytotoxic reactive oxygen species [20]. IL-1β and TNF-α secreted by alveolar macrophages, will prime and amplify the reaction after acute lung injury caused by WLL, induce an inflammatory cascade and worsen lung injury [21–23]. As an important anti-inflammatory cytokine, IL-10 controls inflammation via inhibiting pro-inflammatory mediators [24]. In this study, the serum level of SOD of group L after WLL was significantly increased than group H at T3 and T5. Patients in the group L had more antioxidant products in the serum after surgery, which could reduce the oxidation reaction compared with those in group H. IL-10 in serum at T3 was significantly increased in both groups, while the increase of IL-10 in group L was higher than that in group H. The serum level of MDA was significantly decreased in the group L at T3 compared with group H. These all indicate that low negative suction pressure has a stronger anti-inflammatory effect than high negative suction pressure. However, the serum level of TNF-α was significantly decreased in the group H, which may be due to that the high negative suction pressure during WLL could better clear out the impurities in the patients' lungs, so that the macrophages could reduce the secretion of TNF-α. In the same time, the level of IL-10 and SOD in WLL fluid was significantly increased in the group L. Low pressure of negative suction may suppress the production of reactive oxygen species and be related to its anti-inflammatory properties. It may have a stronger antioxidant effect after surgery, and the probability of acute lung injury is therefore greatly reduced.

The long-term efficacy of WLL in the treatment of silicosis had been initially confirmed, but the long-term efficacy was limited. It was possible that one-time WLL could only play a role of "dust reduction", and the residual SiO_2_ dust in the lungs would continue to damage the lung tissue. In this study, pulmonary function parameters did not become better and even worse than before lavage at one or two months after operation. One WLL may not improve the pulmonary function thoroughly, and many patients need more than once WLL [25].

There are many limitations in this paper. Firstly, only 24 patients were enrolled in the study. No more groups can be divided due to the number of patients were too small. Secondly, there was no normal patient group in the study because normal patients did not need to undergo large volume lung lavage and could not be approved by the ethics committee. We can do animal experiments in the next step to obtain more meaningful data comparison. Thirdly, there was no professional measurement of extravascular pulmonary fluid volume which was an important index of lung edema. Lung edema was not exactly judged only by residual lung fluid volume in this study.

In summary, the above findings point toward the potential benefits of low negative suction pressure as means to suppress oxidative stress, reduce inflammatory cytokine release and decrease lung injury in patients with pneumoconiosis during WLL, but also lead to more residual lung lavage fluid. Despite differing suction strategies, pulmonary function parameters including FEV1%, RV and DLCO% became worse than before WLL.

## Data Availability

The datasets used and/or analyzed during the current study are available from the corresponding author. Not applicable.

## Abbreviations

WLL: Whole lung lavage
ABG: Arterial blood gas
ANOVA: Univariate analysis of variance
BMI: Body mass index
pH: Pondus hydrogenii
PaO_2_: Arterial partial pressure of oxygen
PaCO_2_: Arterial partial pressure of carbon dioxide
LAC: Lactic acid
GLU: Glucose
TNF-α: Tumor necrosis factor-α
IL-1β: Interleukin-1β
IL-10: Interleukin-10
ROS: Reactive oxygen species
MDA: Malondialdehyde
SOD: Superoxide dismutase
MVV: Maximal volumtary ventilation
FVC: Forced vital capacity
FEV1%: Forced expiratory volume in one second
RV: Residual volume
TLC: Total lung capacity
DLCO%: Carbon monoxide dispersion factor
FEV1/FVC%: Forced expiratory volume in one second/forced vital capacity
DLT: Double lumen tube
OLV: One lung ventilation
CPB: Cardiopulmonary bypass
PAP: Polyacrylamide alveolar macrophage
PIP: Peak inspiratory pressure
