# Correction to: Lung injury induced by different negative suction pressure in patients with pneumoconiosis undergoing whole lung lavage

**DOI:** 10.1186/s12890-022-01994-0

**Published:** 2022-05-19

**Authors:** Mingyuan Yang, Baoping Li, Bin Wang, Lei Li, Yurong Ji, Yunzhi Zhou, Rui Huang, Qinghao Cheng

**Affiliations:** 1 Center of Anesthesiology and Pain, Emergency General Hospital, Beijing, 100028 China; 2 Occupation Medicine Department, Emergency General Hospital, Beijing, 100028 China; 3 Department of Obstetrics and Gynecology, Emergency General Hospital, Beijing, 100028 China

## Correction to: BMC Pulmonary Medicine (2022) 22:152 10.1186/s12890-022-01952-w

Following publication of the original article [1], the authors flagged that the article had published with an error in the authorship: Rui Huang and Qinghao Cheng had neither been marked as equal contributors nor as co-corresponding authors. The published article has been updated and the correct authorship may be seen in this correction.

The authors thank you for reading and apologize for any inconvenience caused.

